# The effects of patient characteristics on ADHD diagnosis and treatment: *a factorial study of family physicians*

**DOI:** 10.1186/1471-2296-11-11

**Published:** 2010-02-08

**Authors:** Christopher P Morley

**Affiliations:** 1 Department of Family Medicine, SUNY Upstate Medical University, 750 East Adams St, MIMC Suite 200, Syracuse, NY 13210, USA; 2 Department of Public Health and Preventive Medicine, SUNY Upstate Medical University, 750 East Adams St, Setnor Hall 4th Fl, Syracuse, NY 13210, USA

## Abstract

**Background:**

Attention Deficit Hyperactivity Disorder (ADHD) is a costly and prevalent disorder in the U.S., especially among youth. However, significant disparities in diagnosis and treatment appear to be predicted by the race and insurance status of patients.

**Methods:**

This study employed a web-based factorial survey with four ADHD cases derived from an ADHD clinic, two diagnosed with ADHD in actual evaluation, and two not. Randomized measures included race and insurance status of the patients. Participants N = (187) included clinician members of regional and national practice-based research networks and the U.S. clinical membership of the Society of Teachers of Family Medicine. The main outcomes were decisions to 1) diagnose and 2) treat the cases, based upon the information presented, analyzed via binary logistic regression of the randomized factors and case indicators on diagnosis and treatment.

**Results:**

ADHD-positive cases were 8 times more likely to be diagnosed and 12 times more likely to be treated, and the male ADHD positive case was more likely to be diagnosed and treated than the female ADHD positive case. Uninsured cases were significantly more likely to be treated overall, but male cases that were uninsured were about half as likely to be diagnosed and treated with ADHD. Additionally, African-American race appears to increase the likelihood of medicinal treatment for ADHD and being both African-American and uninsured appears to cut the odds of medicinal treatment in half, but not significantly.

**Conclusions:**

Family physicians were competent at discerning between near-threshold ADHD-negative and ADHD positive cases. However, insurance status and race, as well as gender, appear to affect the likelihood of diagnosis and treatment for ADHD in Family Medicine settings.

## Background

Attention Deficit Hyperactivity Disorder (ADHD) is a wide-spread and behavioral condition in the U.S. It affects roughly 7% to 9% of the U.S. population aged 4-17 years [1-3]. By one estimate, ADHD costs the U.S. economy between $36 billion and $52 billion per year (in 2005 U.S. dollars), or roughly $12,000 to $17,000 per affected individual, per year [4,5]. ADHD is also the source of significant morbidity, including social, emotional, economic, and even secondary physical suffering in affected children. It is associated with an increased propensity for lowered self-esteem, stigmatization, school failure, poor socialization, tobacco use, drug and alcohol abuse, traffic accidents and occupational issues that persist into adulthood [6-10]. However, only slightly more than half of those diagnosed with ADHD are actually under treatment for it at any given time [2,3].

The data are unclear on the precise amount of ADHD diagnosis and treatment occurring in primary care settings, but it is fair to say that it is substantial. In fact, as much as 65% - 85% of initial ADHD diagnosis and treatment occurs in primary care settings (e.g. Pediatric or Family Medicine practices) [11,12]. Once a child has been determined to meet the basic criteria for ADHD, the next step recommended by a number of authoritative sources is referral to an ADHD specialist, such as a mental health professional, ADHD subspecialty clinic, or Neurodevelopmental Pediatrician [13,14]. However, in practice a lack of referral resources, long waiting lists or other accessibility issues at subspecialty clinics, and insurance issues often conspire to leave diagnosis and treatment in the hands of the primary care physician [12,15,16].

Both the American Academy of Child and Adolescent Psychiatry and the American Academy of Pediatrics have issued formal guidelines for the diagnosis and treatment of ADHD [17-19]. However, these guidelines have been difficult to implement, [17,20-22] and a number of voices have stressed the need to improve quality of care for children presenting with ADHD symptoms in primary care settings [16]. Implementation, acceptance, and even knowledge of the existence of ADHD guidelines are all highly variable within and across medical specialties and disciplines [23-28]. Possibly owing to the fact that the present primary care guidelines for ADHD management originated within the Pediatric medical specialty, several of these studies indicate a particular lack of familiarity with the guidelines, and with comfort levels in diagnosing and treating ADHD, in Family Physicians [11,23,27,28]. For example, a study of primary care physicians in Minnesota found that 54% of Family Physicians were altogether unaware of the AAP guidelines introduced in 2004 [29]. Similarly, Rushton and colleagues surveyed 1374 primary care physicians, and found that 91.5% of Pediatricians were familiar with AAP guidelines, while only 59.8% of Family Physicians were similarly familiar [27]. Even more pronouncedly, Lanham and colleagues surveyed 17 civilian & 17 military Family Medicine residency programs, and found that only 22% were familiar AAP guidelines, and only 12.9% reported that they regularly screen for ADHD. Additionally, a majority of respondents across both program types used inappropriate methods to arrive at a diagnosis; 70% of Family Physicians surveyed reported use of a child's behavior in the office in the diagnostic process, and 53% use response to stimulants to diagnose [23].

In addition to apparent variability in clinical practices, a wide array of social factors appear to cause disparities in diagnosis and treatment for ADHD, especially between races, genders, and socio-economic indicators [30-32]. Studies based upon parental reports suggest higher prevalence rates in whites [1], and a study by Froelich et al, comparing prior diagnosis of ADHD to assessment via a standardized instrument in a nationally representative cross-sectional survey, determined that poor children were more likely to meet ADHD criteria (adjusted odds ratio [AOR], 2.3; 95% confidence interval [CI], 1.4-3.9), that 47.9% of children who met criteria actually had a prior diagnosis of ADHD, and that only 32.0% were consistently treated with appropriate medications in the year prior to the survey. The same study found that ADHD tends to be missed in girls, relative to boys, (AOR, 0.3; 95% CI, 0.1-0.8), and wealthy children had a greater likelihood of receiving regular medication than the poorest of the sample (AOR, 3.4; 95% CI, 1.3-9.1). As a corollary to the overarching issue of poverty, other studies looking explicitly at patient insurance status have distinctly identified it as having a significant effect upon who is diagnosed with and treated for ADHD [3,33].

ADHD diagnosis and treatment rates clearly fall along several socio-demographic lines. Gender, geography, wealth, and ethnicity all seem to play roles, in addition to race and insurance status [1,2,30,34-38].

Given the apparent socioeconomic disparities that have been observed in ADHD diagnosis and treatment, as well as the particular issues that appear to be present when cases of suspected ADHD present in primary care offices, a vignette-based factorial web survey experiment designed to test the effects of two social factors on the likelihood of diagnosis and treatment of Attention Deficit Hyperactivity Disorder (ADHD) in children, as applied by primary care providers (PCPs), and focused upon Family Physicians (FPs), was conducted in a convenience sample of PCPs. This study has focused upon race and insurance status in an isolated fashion, as these two items may co-vary [33]. The specific objectives of this endeavor are to test the effect of patient race (black vs. white) and insurance status (insured vs. uninsured) upon likelihood of diagnosis with ADHD by primary care providers (PCPs), particularly Family Physicians (FPs). The central research question addressed by this study asks whether randomly varying the race and insurance status in marginal ADHD cases has any effect upon the likelihood of ADHD diagnosis and treatment.

## Methods

### Factorial Survey Design & Procedures

A more complete description of the methods employed in this study is available elsewhere [39]. However, it is useful to offer an overview of these methods here. The current project utilized four case studies, or vignettes, each consisting of three paragraphs of information about a particular patient. The vignettes were included in a web-based survey of primary care physicians, with each respondent seeing each of the four vignettes which they then "diagnosed" and "treated" by selecting from a set of choices following each vignette. The vignettes in each case were held constant from respondent to respondent, with the exception of the first line of each. This first line was a generic statement following the form:

*"An X-year old **(White/African-American), (privately insured/uninsured) **child is in your office for a visit." *Randomization of race and insurance status was performed via a proprietary randomization engine employed by Grant Systems, Inc., and occurred as the respondent entered each vignette page, resulting in 256 different possible combinations of race/insurance altered vignettes. Following the four vignettes, respondents then answered 20 questions about their training, specialization, practice, and demographic traits, attitude towards behavioral diagnoses in children, and comfort treating childhood behavioral cases. The four cases utilized in this study are presented in Additional File 1.

This study was granted an exemption from review by the Institutional Review Boards of Syracuse University and SUNY Upstate Medical University. Nevertheless, all had to pass through an informed-consent page before participating in the survey. Respondents who chose to continue next went to a page containing basic instructions and a randomly selected version of the first vignette (i.e. White/Privately Insured, White/Uninsured, African-American/Privately Insured, African-American/Uninsured). At page bottom, immediately following the vignette, were three questions, asking the respondent to select a) a diagnosis from a set of four (No Diagnosis, Oppositional Defiant Disorder, ADHD, Bipolar Disorder) and b) a treatment option, from a set of six (No tx/monitoring, psychotherapy only, stimulant, combined medication and psychotherapy, mood stabilizer, anti-psychotic). Once finished with the first vignette and set of questions, respondents were taken to randomized versions of Vignettes 2 through 4, with the same set of questions and options after each.

### Case Development & Validation

The vignettes utilized in this experiment were derived from four cases seen at a local ADHD specialty clinic, with two of the cases nominally meeting criteria for ADHD upon formal evaluation and two cases of suspected ADHD that ultimately did not meet the criteria for diagnosis, upon evaluation. The four cases were intentionally distributed as two sub-threshold cases, 1 male, 1 female, and two "true" ADHD cases, again, 1 male, 1 female. These cases where selected by two psychologists specializing in ADHD diagnosis, who sent case information on anonymous abstracting forms. Information from the abstracting forms was utilized to create a 3-paragraph, narrative vignette describing each case. All four of the cases were derived from actual patients who had been psychometrically tested for the presence of ADHD after referral from an external source. Marginal, as opposed to "clear-cut," cases were used in order to isolate the effects of patient race and insurance status from large variations in presentation of symptoms from case to case.

Some confidence in the content validity of the vignettes employed in this study may be taken from the fact that they were drawn from the facts of actual cases. Nevertheless, additional steps were taken to assure that the vignettes performed reliably when employed within the instrument, i.e. that they produced reasonably predictable diagnostic recommendations when read by multiple clinicians from a variety of relevant specialties. Additionally, the vignettes were modified iteratively until they were both reliable in eliciting appropriate responses from a beta panel of diagnosticians, as well as directionally accurate (i.e. until each vignette elicited consistent diagnoses that matched that of the psychometrically evaluated actual cases they were derived from).

Following the abstraction and vignette generation, each of the four vignettes were submitted to a panel of 18 primary care physicians and mental health experts blinded to the "true" diagnosis in each case in a draft version of the web-based instrument. Each rater selected a diagnosis and treatment from a set of options for each vignette and was asked to provide summary comments about what affected their decision in a text box following each vignette.

The alpha panel test was conducted in May and June of 2008. Results were formally analyzed by rotating the matrix of responses so that each vignette represented a row and each "response" was the diagnosis assigned by each of the 18 respondents. Cronbach's Alpha split-half correlation was calculated for the matrix. Additionally, the comments entered into the text boxes were analyzed for appropriate content, e.g. that respondents were not openly declaring social biases or blatantly incorrect knowledge while diagnosing each case. Finally, panel respondents confidentially made recommendations on improving each vignette either in direct conversation, by e-mail, or via a secondary web interface. A second goal of the alpha test was to assess the pure functionality of the instrument and web interface, including the speed, data collection and delivery mechanisms, randomization engine, password functioning, etc.

The instrument, vignettes, and collection interface were modified based upon initial data analyses, content analysis of remarks included in question #3 after each vignette, direct verbal feedback, and observation of the manner in which the instrument and interface functioned. The second iteration of the survey instrument was successfully beta-tested in August and September, 2008, and produced a reasonable reliability score (Crohnbach's α = .866).

### Sample Size and Recruitment

A pre-study power analysis, performed using G*3 Power 3.0.5 [40], determined that a total N of 129 cases diagnosed would be necessary to detect a moderate effect using four independent variables, with α at .05 (CI = 95%). A need for an N of 129 cases translated to a minimum of 32 respondents, completing four cases each.

Several strategies were utilized to obtain and surpass the target N. First, distribution through regional and national Practice-Based Research Networks (PBRNs) insured that physicians interested in participating in research studies were invited to participate; such interest is an assumed pre-requisite for members of PBRNs. Additionally, going to both regional and national PBRNs, as well as approaching the non-PBRN general membership of professional societies such as the Society of Teachers of Family Medicine, opened the recruitment pool up to literally thousands of individuals. As an incentive for participation, invitees were offered a $25 stipend plus the opportunity to earn free Continuing Medical Education in the future (currently under development).

### Data Analysis

Analyses of the dependent variables were conducted in several steps. Before proceeding to regression analyses, χ^2 ^statistics were computed to determine whether there were any significant differences in the distribution of respondent characteristics between vignettes randomized as White/Insured, White/Uninsured, African-American/Insured, or African-American/Uninsured (the four possible factorial combinations). Absence of significant differences in respondent characteristics across the four factorial combinations was accepted as evidence that the randomization procedure was effective, and hence it was unnecessary to control for any non-significant respondent characteristics.

The data were then analyzed by creating binary diagnostic and treatment outcome variables. For diagnostic outcomes, cases given "no diagnosis" or Oppositional Defiant Disorder (ODD) were labelled 0 and cases given ADHD or Bipolar (BP) were labelled 1. ODD and BP were so infrequently selected that removing them from the analysis made essentially no statistical difference. Similarly, treatment selections were grouped into those that would result in a medicinal intervention (1, where stimulant, combined therapy, mood stabilizer, or anti-psychotic were selected), and those that would not (0, where No treatment or psychotherapy only were selected). Binary logistic regressions were calculated for each dependent variable (Diagnosis & Treatment) using the responses from the four cases, modelled against race, insurance status, case gender, ADHD status of the case, and interaction effects.

## Results

### Sample Characteristics

Collection of responses for the full data set (N = 187) occurred primarily from September through December, 2008, with a small number of late respondents entering the survey in January and early February, 2009. All respondents completed the survey. Respondents came from 35 states, with the largest number coming from New York (roughly 30%), followed by Georgia (roughly 9%) and Pennsylvania (roughly 7%). The sample was heavily skewed towards White respondents (87.7% White) and was evenly split between male and female respondents (50.8% male, 49.2% female). Physicians educated in the United States were also over-represented at 94.6%, compared to the population of total U.S. physicians (74.5%) [41] and to Family Physicians (83.7%) [42].

172 of 187 respondents identified themselves as practicing within the Family Medicine specialty, with the rest identifying themselves as Pediatricians. Respondents were mostly MD's (86.6%), followed by DO's (11.2%), with one physician respondent holding a non-U.S. medical credential (M.B.B.S). Three mid-levels (Nurse Practitioners or Physician Assistants) also responded. Average time in practice was approximately 16 years and was fairly evenly distributed across a range of 1 to 36 years (S.D. = 9.71 years). Most respondents saw at least some poor and/or non-white patients in their practices, with 39% indicating their patient panel consisted mostly of poor/non-white or mostly poor/white patients. An additional 17.1% selected an "Other" option to describe their patient population but the majority of these respondents textually indicated a diverse patient population that included poor patients, non-white patients, or both. Almost two thirds of respondents indicated either Urban, Suburban, or mixed Urban/Suburban practice settings (64.1%). The full distribution of sample characteristics is displayed in Tables 1, 2 and 3.

**Table 1:** Distribution of Respondents across U.S. States

*State*	*Frequency*	*Percent*
New York	55	29.41%
Georgia	16	8.56%
Pennsylvania	13	6.95%
California	9	4.81%
Michigan	9	4.81%
Illinois	8	4.28%
Missouri	8	4.28%
Virginia	8	4.28%
New Jersey	7	3.74%
North Carolina	6	3.21%
Texas	5	2.67%
Indiana	4	2.14%
South Carolina	4	2.14%
Wisconsin	4	2.14%
Arizona	3	1.60%
Massachusetts	3	1.60%
Minnesota	3	1.60%
Maine	2	1.07%
Mississippi	2	1.07%
Ohio	2	1.07%
Wyoming	2	1.07%
Alabama	1	0.53%
Alaska	1	0.53%
Colorado	1	0.53%
Delaware	1	0.53%
Hawaii	1	0.53%
Louisiana	1	0.53%
Maryland	1	0.53%
Montana	1	0.53%
Nevada	1	0.53%
New Mexico	1	0.53%
Oklahoma	1	0.53%
Oregon	1	0.53%
Tennessee	1	0.53%
Washington	1	0.53%

**Total**	**187**	**100.0**

**Table 2:** Demographic Characteristics of Respondents

	Number	Percent	Population
**Gender**			
*Male*	*95*	*50.8%*	76.0%^/64.9%*
*Female*	*92*	*49.2%*	24%^/35.1%*

	**187**	**100%**	

**Native U.S**			
*U.S*	170	91%	81%^
*Non-U.S*	17	9%	19%^

	**187**	**100%**	

**Race**			
*White*	164	88%	44-86%'
*Asian/Pacific Islander*	9	5%	8%'
*Black*	4	2%	2%'
*Other*	10	5%	~2%'

	**187**	**100%**	

**Hispanic/Latino**			
*Non-Hispanic*	179	95.7%	98%'
*Hispanic/Latino*	8	4.3%	3%'

	**187**	**100%**	

**Medical School**			
*Attended U.S. Medical School*	177	94.6%	74.5%^/83.7%*
*Attended non-U.S. Medical School*	10	5.4%	25.5%^/16.3%*

	**187**	**100%**	

**^ **Total U.S. Physician Population, as of 2000 (American Medical Association.) http://www.ama-assn.org/ama1/pub/upload/images/373/internettable.gif

* Total membership of American Association of Family Physicians as of 2008 (American Academy of Family Physicians.) http://www.aafp.org/online/en/home/aboutus/specialty/facts/2.html

'Distribution of Nonfederal Physicians by Race, 2008, Kaiser Family Foundation. http://www.statehealthfacts.org/comparetable.jsp?ind=431&cat=8

**Table 3:** Practice Characteristics of Respondents

	Number	Percent
**Credential**		
*MD*	*162*	*86.6%*
*DO*	*21*	*11.2%*
*MBBS*	*1*	*0.5%*
*Mid-Level*	*3*	*1.6%*

	**187**	**100%**

**Specialty or Area of Practice**		
*Family Medicine*	*172*	*92.0%*
*Pediatrics*	*15*	*8.0%*

	**187**	**100%**

**Years in Practice**		
*Range: 1-36 years; Weighted Avg: 16.09 years (SD: 9.71)*		

**Patient Population**		
*Mostly poor, non-white*	*52*	*27.8%*
*Mostly poor, white*	*21*	*11.2%*
*Middle class, mixed race/ethnicities*	*57*	*30.5%*
*Middle class, mostly white*	*24*	*12.8%*
*Middle class, mostly white*,	*1*	*0.5%*
*Other (Most indicated low SES)*	*32*	*17.1%*

	**187**	**100%**

**Practice Setting**		
*Rural*	*22*	*11.8%*
*Mixed Rural/Suburban*	*28*	*15.0%*
*Mixed Suburban/Urban*	*24*	*12.8%*
*Mixed Rural/Suburban/Urban*	*12*	*6.4%*
*Mixed Rural/Urban*	*5*	*2.7%*
*Suburban*	*32*	*17.1%*
*Urban*	*64*	*34.2%*

	**187**	**100%**

### Results for Diagnostic & Treatment Decisions

Several exploratory analyses that incorporated respondent characteristics as covariates, including type of professional credential, level of training, normal patient mix (peds/adult and race/class), and practice setting (along a rural-urban scale) did not reveal any of these covariates to be statistically significant. Of possible note, years of training appeared to approach significance, and so number of years in practice was included in the final binary logistic regression models.

Neither race nor insurance status appears to be an independent predictor of diagnosis in the clustered, four-case model presented in Table 4. The most pronounced effect was that of "true" ADHD status of the vignette: the ADHD-positive cases both produced relatively large effect sizes and low p-values when regressed against diagnosis, indicating that ADHD status was a primary endogenous predictor of diagnosis, with the male ADHD-positive case almost three times as likely to be diagnosed (Case 3 [Male, Positive]: OR = 8.006, p < .001; Case 4 [Female, Positive]: OR = 2.967, p < .001). Insurance status, however, appears to interact with gender to influence diagnosis in this model. Male vignettes randomized as uninsured cut the odds of diagnosis in half, although this effect did not achieve statistical significance (O.R. = .546, p = .062).

**Table 4:** Binary Logistic Regression results of the four-case model.

Variable	Diagnosis 0 = no Dx, or ODD 1 = ADHD or BP *Odds Ratio (p), CI*	Treatment 0 = No Tx, or Psychotherapy Only 1 = Medicinal or Combined Tx *Odds Ratio (p), CI*
African-American (AA)	1.343 (0.191), 0.863 - 2.092	1.514 (0.072)^, 0.963 - 2.380
Uninsured	1.176 (0.529), 0.710 - 1.947	1.837 (0.021)*, 1.095 - 3.081
AA × Uninsured	0.700(0.269), 0.372 - 1.318	0.582 (0.110)^, 0.303 - 1.117
Case 2	1.398 (0.197), 0.841 - 2.326	0.876 (0.595), 0.519 - 1.478
Case 3	8.006 (<.001)**, 4.936 - 12.987	11.840 (<.001)**, 7.090 - 19.772
Case 4	2.967 (<.001)**, 1.751 - 5.028	2.950 (<.001)**, 1.734 - 5.017
Male(1) × Uninsured(1)	0.546 (0.062)^, 0.289 - 1.030	0.409 (0.007)**, 0.212 - 0.786
Years in Practice	0.985 (0.060)^, 0.969 - 1.001	0.986 (0.106), .970 - 1.003
Constant	0.832 (0.061)	0.651 (0.102)

* Significant at the .05 level.

**Significant at the < .01 level

^Approaching significance

The effects of race and insurance status upon treatment decisions were more direct. Cases randomized as African-American were slightly more likely to be medicated, an effect which approached but did not reach significance (O.R. = 1.514, p = .072), and being uninsured significantly increased the likelihood of being medicated (O.R. = 1.837, p = .021). Cases randomized as both African-American and uninsured were less likely to be medicated, although this effect was not significant. As with diagnostic decisions, treatment decisions were highly predicted by true ADHD status of the cases. Furthermore, a gender contrast once again appears between the ADHD-positive cases, with Case 3 (the male case) being over 11 times more likely to be medicated or treated with combined medication and psychotherapy (O.R. = 11.840, p < .001) than the male, ADHD-negative reference case, while Case 4 (the female case) was only about 3 times as likely than the reference case to be medicated (O.R. = 2.950, p < .001).

## Discussion

This study was a focused exploration into how primary care physicians, and specifically Family Physicians, approach the diagnosis and treatment of ADHD in children. It was specifically looking at how social factors may influence an ostensibly objective clinical process. Given this central focus, it is important to begin this discussion by clearly stating that the respondents, as a whole, were effective at correctly discriminating between ADHD and non-ADHD cases in their diagnostic decisions. ADHD-positive cases were significantly more likely to be diagnosed and treated in all models constructed with these data. Furthermore, the cases that study participants were reading and responding to were intentionally marginal; all displayed at least some symptoms consistent with an ADHD diagnosis. Also, the participants in the study did not have the opportunity to utilize follow-up questions, formal diagnostic processes (such as real-time interviews with parents, or evaluation via Connors instruments, etc.) or consults, or otherwise acquire additional case information, as they would in an actual clinical setting. Despite the uncertainty and unfamiliarity with ADHD guidelines identified by other studies in primary care and FP populations [11,23,24,29,43], the respondents to this survey were, as a group, competent at handling ADHD cases. However, insurance status and race appear to play a role in how ADHD is diagnosed and, more pronouncedly, treated, in primary care.

### Effects of Insurance Status

Insurance status appears to play a somewhat complicated role in determining which children are diagnosed with and treated for ADHD in a primary care setting. Being uninsured lowered the odds of diagnosis in male cases, an effect that comports with trends repeatedly observed elsewhere [33]. This diagnosis-suppressing effect of being identified as uninsured was not observed in the female-identified cases. The question requires further study, however. What is clear from this study is that, at least in male cases, being uninsured reduced the odds of diagnosis.

The picture is somewhat different for treatment decisions. Both African-American race and uninsured status individually increase the odds of medicinal or combined medicinal/psychotherapeutic treatment, with the insurance effect being significant, and the race effect approaching significance. However, when interacted, the two variables together serve to cut the odds of treatment in half, although this interaction effect was not significant in this relatively small study. Also observed was a very significant interaction between male gender and being uninsured, with this combination of factors reducing the odds of treatment by half, in line with the observed effect on diagnosis. It would be speculative to suggest explanations for this effect at this point, but this finding underscores the need for further study of gender and ADHD. The addition of both gender-null and insurance-null cells in a future follow-up study would help resolve this question.

Fundamentally, what may be drawn from the current study about insurance status is that patients presenting as uninsured are generally less likely to be diagnosed with and treated for ADHD. This effect is apparently moderated by both race and gender. These latter interactions require further examination, however.

### Effects of Race

Race was a non-factor in predicting diagnostic outcomes in this study. However, it appears to play a more important role in determining physician decisions about treatment, as well as in moderating the effect of insurance status. As noted above, the positive effect observed in the female ADHD positive case when presented as uninsured was completely and significantly reversed when the case was both African-American and uninsured. When grouped by ADHD status, the ADHD-positive cases were more than twice as likely to be diagnosed when identified as African-American, but only about a third as likely to be diagnosed when identified as both African-American and uninsured. This indicates that some but not all of this effect may be due to its presence in Case 4. While neither race alone nor the race × insurance interaction were statistically significant in the four-case model, the trends remained consistent - African-American race alone increased the odds of treatment, but the interaction between race and insurance status decreased these odds. The fundamental role of race appears to moderate the effects of insurance status.

### Weaknesses of the Study

There were several central challenges in the design of this study. First was the need to generate an adequate sample size. The study population consisted of busy, over-surveyed primary care physicians. Physician surveys have notoriously low rates of response, and the methods that prove most effective - monetary incentives and mixed web/paper survey distribution - are generally costly and labor-intensive [44,45]. Given limited resources, providing both an adequate monetary incentive and pursuing a mail-based paper alternative to a web-based survey was impractical. Additionally, the web survey was constructed to randomize both race and insurance status of the vignettes as respondents entered the instrument; the use of a paper survey would have required pre-randomized blocks of invitees to receive fixed paper versions of the survey. While this is not theoretically problematic, the low response rates typically seen in physician surveys raised the danger of an imbalanced response rate between blocks. While such an eventuality is correctable with continued collection focused upon the under-represented blocks, such a strategy would have demanded more resources than were available. For these practical reasons, this study relied upon web-based collection alone.

An obvious challenge for this study was the fact that the respondents were reacting to purely textual vignettes, as opposed to live, actual or standardized patients. Despite this weakness, vignette-based studies have a long history of effectively demonstrating the effects of discrete factors while holding a body of information constant. For example, Lutfey, McKinley and colleagues have recently utilized vignettes to examine both the cognitive pre-dispositions of physicians as they gather patient information, as well as the resulting decisions of such cognitive processes. For example, Lutfey and colleagues recently used a vignette based approach to do precisely this for clinical decision making scenarios involving Coronary Heart Disease and Depression [46-49]. Another related challenge in pursuing clinical vignette research is the basic need to create vignettes and response options that are realistic enough to be taken seriously, but not so complex as to hinder efficient and interpretable analysis. In the current context, physicians are not faced with a simple choice of deciding "ADHD or not," in binary fashion, when evaluating a child with behavioral symptoms. Offering a diagnostic choice between ADHD or no diagnosis at all would have been unrealistic, and would furthermore have partially revealed the study design to respondents. Therefore, reasonable differential diagnoses were offered along with the choice between selecting ADHD or no diagnosis at all.

Additionally, the physician sample obtained for this study was clearly not representative of the U.S. physician population as a whole. Recruitment relied in some cases upon pass-along invitations by network directors, as well as upon snow-ball techniques from colleague to colleague. An accurate response rate is therefore difficult to measure. It is safe to say, however, that the response rate fell below 10%. This study therefore relied upon a sample of convenience. It furthermore relied upon those who were willing and able to open and respond to an e-mail invitation and web-based survey instrument.

There were pragmatic decisions made regarding the structure of the data matrix that may be viewed as study weaknesses as well. Given the extreme difficulty in recruiting large samples of physicians to complete surveys, the use of four vignettes per respondent allowed for the potential to quadruple the power of the study, and this proved crucial. However, the use of four cases per respondent introduced the issue of panel-oriented autocorrelation in the variance-covariance matrix. This proved to be of only moderate concern, and was generally addressable by incorporating between-subject and between-case variables into the analytic models, obviating the need for more advanced statistical measures which dilute the efficiency or interpretability of results to some extent. Some issues could not, however, be addressed. For example, the ability to analyze the interaction between clinician and patient race was severely limited by multicollinearity, and clinician/patient gender interactions were severely penalized by the data structure as well. Both had to be eliminated altogether. Additionally, it must be noted that the truly experimental phase of this study is limited to the analyses of the individual cases, which may have been underpowered. Although useful, the two-case and four-case analyses combined multiple vignettes, thus eliminating the ability to control for all variation other than race and insurance status. These analytic phases must therefore be considered quasi-experimental.

## Conclusions

Presently, reform of the U.S. healthcare system is under robust and serious discussion. At the core of this discussion is a presidential plan to reduce health disparities, drive down healthcare costs, increase usage of electronic medical records, and universalize health insurance, improve coordination of care, and enhance quality of care [50]. In such an expansive conversation, this study serves to illustrate that disparities in healthcare must remain central, and that the root causes require further exploration. It is no secret that disparities in health and healthcare exist, nor that differentiated patient characteristics such as race, insurance status, and others are intimately connected to such disparities, for a variety of conditions and settings [51-54]. This study serves to illustrate one manner of how patient insurance status and race may lead to a disparity in the care patients receive across groups, in the context of one condition.

Several recommendations along these lines may be made based upon the current study. First, further development and evolution of guidelines for the diagnosis and treatment of ADHD [16,22,27,55,56] need to incorporate a) explicit statements regarding an emphasis on impairment and, to a lesser extent, upon symptom presence, and b) explicit statements directing physicians to avoid relying upon socioeconomic or racial characteristics when conducting evaluations. Future studies may implicate the need to include gender in this list. Alternatively, the next iteration of clinical guidelines may be adopted to more closely resemble the usual case presentations of females and non-white patients. In particular, some have criticized current and previous clinical guidelines for ADHD as being more applicable to boys than girls, and developing guidelines that are gender-sensitive may be a crucial next step [57]. As noted previously, there are noted gaps in ADHD guideline familiarity within the community of FP's. Guidelines that can realistically be applied must not only be developed [15,26]; it is important to proactively disseminate such information to all who would make use of it.

Additionally, as noted by Starfield [58], the process of referral must be streamlined. In this case, the specific referral path under consideration is from primary care to mental health specialist. While the primary care physicians who participated in this study were generally effective at correctly identifying and treating ADHD, easier access to specialist services may serve to ameliorate disparities by facilitating more extensive diagnostic evaluations, especially in ambiguous cases. A lack of referral options has been noted as a barrier to care in a number of studies of the ADHD diagnostic process in the U.S [16,59].

Given both the results and the weaknesses described above, the first and foremost recommendation is that additional research is needed. Specifically, an expansion of the vignette-based approach is in order, which should have the following aims:

1. Incorporation of gender as a factorial variable, along with race and insurance status

2. Increase in study N

3. Increase inclusion of Pediatricians

4. Increase inclusion of non-Academics.

Additionally, follow-up studies incorporating data from actual practice should be conducted, to triangulate and corroborate the findings presented here. Ultimately, a multi-method approach employed across convergent studies will be necessary and is warranted. Finally, utilization of the methods employed in this study to examine the effects of patient characteristics in other medical and mental health specialties, as well as in educational professionals, is warranted.

What this study has examined is but one very particular context where disparities in the provision of healthcare, and specifically the diagnosis and treatment of ADHD, may be generated. Clearly, there is much more work to be done in examining other sources of disparity. However, what may be determined from the results presented here is that insurance status clearly impacts the provision of ADHD services in primary care. Ameliorating health insurance disparities, and improving guidelines to enhance their applicability to uninsured, referral-deprived, and non-white/non-male patients are one step toward accomplishing an egalitarian distribution of services for ADHD in children.

## Competing interests

The author declares that they have no competing interests.

## Authors' contributions

CPM designed the project, conducted all procedures and analyses, and wrote the text.

## Pre-publication history

The pre-publication history for this paper can be accessed here:

http://www.biomedcentral.com/1471-2296/11/11/prepub

## Supplementary Material

Additional file 1 **ADHD Vignettes used in this study**. Vignettes 1 & 2 were designed to be sub-clinical, non-ADHD, and were derived from true cases where ADHD was ruled out upon a full evaluation of the patient. Vignettes 3 & 4 were derived from cases where a full evaluation led to a diagnosis of ADHD. Click here for file
