# Supplementary material for: The effects of patient characteristics on ADHD diagnosis and treatment: a factorial study of family physicians
Source: BMC Fam Pract. 2010 Feb 8;11:11. doi: 10.1186/1471-2296-11-11 (PMC2828998; doi:10.1186/1471-2296-11-11)
Supplement: Additional file 1 — ADHD Vignettes used in this study. Vignettes 1 & 2 were designed to be sub-clinical, non-ADHD, and were derived from true cases where ADHD was ruled out upon a full evaluation of the patient. Vignettes 3 & 4 were derived from cases where a full evaluation led to a diagnosis of ADHD. [file 1471-2296-11-11-S1.DOC]

**Additional File 1 - ADHD Vignettes used in this study**

Vignettes 1 & 2 were designed to be sub-clinical, non-ADHD, and were derived from true cases where ADHD was ruled out upon a full evaluation of the patient. Vignettes 3 & 4 were derived from cases where a full evaluation led to a diagnosis of ADHD.

**Vignette 1:** derived from the case of an 8-year old male who, upon formal evaluation at an ADHD specialty clinic, was not diagnosed with ADHD

An 8-year old African-American, privately insured male child is in your office for a visit to follow up on some behavioral issues noted by a teacher. In the spring prior to this appointment, the patient’s’ teacher rated problems in the clinical ranges for social difficulties, thought problems, attention problems, and aggressive behavior. That teacher also noted that the patient becomes easily upset and had difficulty settling down, exerting self control, and anticipating consequences. During the parent interview, the parents indicated that the patient needs redirection, especially in large group settings, and can be disruptive, impulsive, inattentive, and challenged when he has to stay still for any period of time. Furthermore, the parents indicate that the patient can feel rejected by peers, although they suspect this has more to do with sensitivity on the child’s part. While the disruptiveness and need for redirection has at times led to some chiding by peers, the patient clearly reciprocates emotions appropriately with parents and other family members.

The child has previously been diagnosed as having Pervasive Developmental Disorder-Not Otherwise Specified (PPD-NOS) when he was three because of extremely uneven social skills and development. This diagnosis was made by the child’s pediatrician at the time, although there is no record of formal assessment. Since that time he has made substantial gains in those arenas, especially once his parents collaborated on a behavioral plan. Although he does not have many friends, he does have two boys he plays with both at home and school. While the prior year’s teacher was concerned about his behavior, the boy was never was so poorly behaved that he was unable to learn; the child’s grades have always been average.

Because the new school year had started just two weeks prior to the appointment, he is presently adjusting to the new teacher, and it is going well by all reports. In general, everyone (including the child) seemed to indicate that, while his behavior was still somewhat problematic, he had made substantial progress over recent years. In addition, the parents have recently divorced and the child indicates during the visit that he no longer witnesses domestic violence. He suggests that his home situation has been much calmer for him over the past 3-4 months, stating that he is “happier now.” During the office visit, the patient seems very nervous, possibly distracted, and fidgets with his hands throughout. Finally, it was determined that the services he was receiving because of the PDD diagnosis, especially the opportunity for brief contact with a resource room teacher, was helpful to him, although the parents were unsure that they were still necessary for him to progress.

______________________________________________________________________________

**Vignette 2:** derived from the case of a 9-year old female child who, upon formal evaluation at an ADHD specialty clinic, was not diagnosed with ADHD

A 9-year old white, privately insured female child is in your office for a visit to follow up on behavioral issues noted by school professionals. Specifically, her 2nd grade school teacher and school psychologist had previously indicated that the child displays difficulty with transitions, is frequently off-task, appears distracted by “internal thoughts,” and struggles with completing assignments. On the Conner’s Teacher Rating Form (TRF), the 3rd grade teacher noted the child is inattentive, fails to finish what is started, and daydreams much of the time. Furthermore, the 3rd grade teacher reports the child occasionally seems easily distracted and is poorly organized. However, the 3rd grade teacher did not report any problems at a significant clinical level on the TRF, and the child’s grades are generally above-average. The teacher reported that the child receives preferential seating near her, is provided with a distraction-free quiet space to complete work, gives her deadlines, breaks down steps, and provides a reminder checklist.

The parents also reported that she is easily distracted, forgetful, and inefficient in completing tasks. They stated their child requires frequent reminders and prompting on simple duties in the home. They reported she struggles with timed tests and has difficulty with transitions. The parents noted that she becomes easily agitated, upset or overwhelmed and at home, prefers considerable guidance to complete homework and other tasks. On the Child Behavior Checklist (CBCL) previously administered to both parents, the father did not report clinical significance on any symptom. The mother reported anxiety/depression, general affective problems, and somatic issues on the CBCL. However, the child has a number of friends that she regularly plays with, and generally demonstrates good emotional reciprocity with family and peers.

The child herself reports that she occasionally has difficulty sustaining attention most particularly in math, and occasionally in other subjects. The child indicated that she is able to pay attention during tasks she enjoys and described feelings of anxiety regarding tests, particularly in math.

______________________________________________________________________________

**Vignette #3:** derived from the case of an 8-year old male who, upon formal evaluation at an ADHD specialty clinic, was diagnosed with ADHD

An 8-year old white, privately insured male child is in your office for a visit after a specific recommendation from his teacher that he be seen by his doctor for follow up on behavioral issues. The patient’s teacher reports that the child is easily distracted, loud and boisterous, fidgety and squirmy, makes careless mistakes. He is also very disorganized, and often forgets to hand in homework even though it is completed. The teacher’s impression is that the child does not seem to listen, generally seems “spacey” and daydreams often, and constantly yells out answer in class without raising his hand. The child’s grades, while not “failing,” are consistently below average.

The parents have reported that the patient’s room is very messy, that he cannot sit still even for 10 minutes, and is impatient; they include that he “has to be first at everything.” They state that he avoids homework, and that his backpack, like his room, is “a disaster.” The parents further describe that he leaves books necessary for homework at school, loses items of clothing like gloves, glasses, hats, or even jackets, several times per year. Finally, they mention that their son’s “personality makes it difficult for him to make friends, or to keep them,” and indicate that this is a constant source of stress. He reciprocates emotions with friends and family, and other than the reported impatience and impulsivity, the child is not overly aggressive or violent. There is no evidence whatsoever to indicate the child is hallucinating, delusional, or having racing thoughts, and there are no obvious recent stressors that might be associated with the disorganization or impulsivity. In fact, the parents note that the child’s behavior has been consistent since he was a toddler.

Other pertinent history includes the fact that, although the child was born full-term, the prenatal course was complicated by maternal smoking. Developmentally, his medical record reports that he has obtained all milestones in an age appropriate fashion, although he receives speech/language therapy once a week for mild speech articulation difficulties. Overall, his health history is rather benign; other than asthma medications, he is not presently prescribed any psychotropic medications.

______________________________________________________________________________

**Vignette 4:** derived from the case of an 11-year old female who, upon formal evaluation at an ADHD specialty clinic, was diagnosed with ADHD

An 11-year old African-American, privately insured female child is in your office for a visit to follow up on behavioral issues noted by a teacher. Her 6th grade teacher reports that she is “quiet…a daydreamer,” and often “seems lost in a fog.” Specifically, the teacher describes that the patient generally stares blankly, is a sluggish or slow thinker, and is routinely spacey, squirmy, fidgety, and restless. The teacher also reports that the child is impulsive, and when she does engage, she will do so in a disruptive manner, blurting out answers. The patient’s grades, although in the “B” range and hence not necessarily bad, are below what the current and most former teachers believe to be her potiential. Both the teacher and the mother agree on this point; upon query, you find the child seems to feel the same way – that she could “do better if she could concentrate”

The patient’s parents are divorced; the divorce occurred when she was 4, and she currently lives with her mother, seeing her father every other weekend. The mother reports that the patient avoids doing homework, is very disorganized, does not seem to listen when spoken to, and has difficulty following directions. The mother is quick to point out that her daughter seems to want to follow directions, but has particular difficulty completing multi-step tasks. The child is definitely very “bright,” a fact on which both teacher and parent concur. This is further reflected in her conversational level with you during the office visit. Furthermore, she reciprocates emotions adroitly. However, despite the fact that she “has good ideas,” according to the mother, she “can’t seem to get them on paper.” She does not follow through on tasks, is in “constant motion” with “some part of her body always moving” according to the mother. You observe this behavior clearly during the office visit. However, the child has been seen several times over the years for these issues, according to her chart, but has not received a formal diagnosis. Previous examination has ruled out neurological disorders, and there is no evidence the child has ever had a seizure of any kind.

The patient herself describes having some difficulty making friends, but does describe having a few “good friends,” a fact corroborated by the mother. Also apparent are generally good social skills, despite the constant fidgeting and apparent attention-shifting around the room. Upon asking about these behaviors, the mother indicates that the patient has “always been that way, since she was little.” One issue that the patient brings up is that she feels “really bad” about how she can’t always “get things done the right way,” and that she knows she can get better grades in school; she just can’t seem to “get it all done.” The mother, teacher, and child all seem to agree on all points presented above.
